# Tattoos and cutaneous squamous cell carcinoma: a population-based case-control study

**DOI:** 10.1007/s10654-025-01230-z

**Published:** 2025-04-25

**Authors:** Emelie Rietz Liljedahl, Malin Engfeldt, Kari Nielsen, Anna Jöud, Christel Nielsen

**Affiliations:** 1 Division of Occupational and Environmental Medicine, Department of Laboratory Medicine, Lund University, Lund, Sweden; 2 Department of Occupational and Environmental Medicine, Region Skåne, Lund, Sweden; 3 Department of Dermatology, Skåne University Hospital, Lund, Sweden; 4 Dermatology, Department of Clinical Sciences, Lund University, Lund, Sweden; 5 Department of Clinical Sciences, Lund University, Lund, Sweden; 6 Clinical Pharmacology, Pharmacy and Environmental Medicine, Institute of Public Health, University of Southern Denmark, Odense, Denmark

**Keywords:** Tattoo, Keratinocyte cancer, Non-melanoma skin cancer, Risk factors, Lifestyle factors, Health risks

## Abstract

The prevalence of tattoos in western countries is about 20%. Tattoo ink may contain carcinogenic compounds. The aim of this study was to investigate if tattoo exposure is associated with an increased risk of cutaneous squamous cell carcinoma in individuals. In this population-based case-control study, 2857 cases aged 20 to 60 years, diagnosed between 2014 and 2017, were identified in the Swedish Cancer Registry. Statistics Sweden identified 3 random age- and sex-matched controls per case from the Swedish Total Population Register using incidence-density sampling. In 2019, participants answered a questionnaire regarding lifestyle factors, including tattoos and sun habits. We used logistic regression to investigate if tattoo exposure was associated with the relative risk of cutaneous squamous cell carcinoma. 61% of the cases and 53% of the controls replied to the questionnaire. Among the 1600 cases and the 4551 controls that participated, 15.1% and 17.6% had at least one tattoo before the index date. We found no increased risk of cutaneous squamous cell carcinoma in tattooed compared with non-tattooed individuals (incidence rate ratio, 0.95; 95% confidence interval, 0.78–1.15). Tattoo exposure was not associated with the risk of cutaneous squamous cell carcinoma in this first study of the association. However, more epidemiologic studies are needed before consensus regarding a lack of association can be reached.

**Supplementary Information:**

The online version contains supplementary material available at 10.1007/s10654-025-01230-z.

## Introduction

Cutaneous squamous cell carcinoma (cSCC) is the second most common cancer in Sweden [1, 2], and the second most common skin cancer type in the United States [3]. The incidence has been increasing steadily since the 1980s. In Sweden, the incidence of primary cSCC was 97 per 100,000 individuals in 2021 [2] compared with around 30 per 100 000 in the early 2000s [4]. Suggested explanations for this increase include better access to care, changing sunbathing habits, and an aging population. Lifestyle-related risk factors are less well studied.

The prevalence of tattoos was recently reported to be > 20% in some European countries, and > 30% in the United States and numbers are reported to be even higher in younger age groups [5–9]. Possible evidence for having a cosmetic tattoo at the site of an early onset basal cell cancer tumour compared to other sites was observed in an American study [10]. Yet, the potential association between tattoos and cSCC has never been investigated.

Traditionally, the colour in tattoo ink comes from various pigments such as azo pigments, carbon black and metal salts, and may contain possible and probable human carcinogens such as polycyclic aromatic hydrocarbons [11, 12], aromatic amines [13–16], and metals [17–21]. In a recent study, the authors showed that the declaration of ingredients in tattoo ink was often incorrect [22]. Another study found forged labels on tattoo ink bottles; permitted pigments were declared instead of the banned ones present in the ink [23]. Risk analysis or testing for the safety of lifelong intradermal exposure to tattoo ink has not been performed, and the long-term health implications are broadly unknown. However, recent evidence suggest that tattoo exposure may be associated with increased cancer risk [24, 25].

Here, we hypothesize, based on the presence of carcinogenic compounds in tattoo ink and the local exposure and potentially systemic exposure over time, that tattoos may be a risk factor for cSCC both locally and systemically, possibly through direct and indirect mechanisms such as DNA-adduct formation and by contributing to an inflammatory environment [26].

The aim of this study was to investigate if tattoo exposure was associated with an increased risk of cSCC. We have included cases aged 20–60 years when diagnosed with cSCC, because the prevalence of tattoos is higher in younger people.

## Materials and methods

### Setting

We aimed to investigate if tattoo exposure is associated with an increased risk of cSCC. Using a population-based case-control design, we identified cases of cSCC diagnosed between 2014 and 2017 in the Swedish National Cancer Register. Controls were identified in the Total Population Register using incidence-density sampling matching on age and sex. We assessed self-reported exposure using a structured questionnaire distributed by Statistics Sweden between February and April 2021. In a case-control study with incidence-density sampling, the odds ratio offers an unbiased estimate of the incidence rate ratio (IRR), which is interpreted as a relative risk [27]. This study was approved by the Swedish Ethical Review Authority (2019–03138).

### Participants

The inclusion criterion was a primary diagnosis of cSCC at the age of 20–60 years. The most recent data at the time of sampling (in 2019) was used, and resulted in cases diagnosed between 2014 and 2017. The date of diagnosis was the index date. The next-of-kin of deceased individuals on January 2021 were contacted according to a prespecified hierarchy (i.e. spouse, common-law spouse, child, parent or sibling) to avoid survivorship bias in case tattoos were associated with a more lethal form of cSCC.

Three random controls of the same age and sex per case were identified by Statistics Sweden using incidence-density sampling. Controls were all free of cSCC at the index date, but individuals with other malignancies were eligible for inclusion. Individuals with psychiatric disorders (diagnosis codes F20-29 in the International Statistical Classification of Diseases and Related Health Problems, 10th Revision) were excluded due to the risk of psychological distress from participation by cross-referencing the study population with the National Patient Register.

Statistics Sweden performed a descriptive dropout analysis on aggregated data regarding the sociodemographic characteristics of responders and non-responders at the time of the survey. Participants were informed that they consented to participate in the study by answering the questionnaire. Next-of-kin were first given information about the study and asked to consent to participate via post, after which they received the questionnaire.

### Variables

#### Outcome: cutaneous squamous cell carcinoma

Incident primary cSCC was defined according to the ICD-O-3.2 (International Classification of Diseases for Oncology, 3rd Edition, 2nd Update) typography code C44 (i.e. non-melanoma skin cancer) in combination with any of the morphology codes listed in Table S1.

#### Exposure: tattoos

We defined exposure as permanent tattoos acquired for decorative, cosmetic, or medical purposes. Cosmetic tattoos entail permanent or semi-permanent makeup, and medical tattoos are used in procedures such as breast reconstruction after mastectomy. Respondents were asked to include tattoos that had been removed; the age they got their first tattoo; and participants were classified as unexposed if they had their first tattoo after their index date.

We powered the study to assess tattoo exposure as the presence or absence of tattoos (dichotomous variable). We also assessed the total area of the body covered with tattoos to investigate crude dose-response relationships by asking participants to estimate the area of tattooed body surface on a categorical scale (< 1 palm/1–5 palms/>5 palms), as well as number of received tattoo sessions (1; 2–3; 4–5; 6–9; >=10). We asked participants to indicate the anatomic location of their tattoos on a body manikin and about the colours used in their tattoos (black, grey, brown, red, blue, green, yellow, white, purple, pink, orange, turquoise, skin colour, or other).

#### Confounders and effect modifiers

Using a directed acyclic graph (dagitty.net, v3.0), we visualized the relationships between exposure and outcome to identify potential confounders (Figure S1). Based on our assumptions, UV exposure and socioeconomic status (SES) constitute the minimal sufficient adjustment set to control for confounding in the relationship between tattoos and cSCC.

The most established risk factor for cSCC is cumulative sun exposure. Low-grade, long-term sun exposure is well described in the literature [28, 29]. We assume that UV exposure may confound the association between tattoo exposure and cSCC through an unmeasured common cause reflecting a general interest in one’s appearance. In addition, we anticipated that UV exposure may be an effect modifier of the association between tattoos and cSCC because UV exposure can induce the release of potentially carcinogenic aromatic amines from azo pigments in the tattoo [15, 16]. We used self-reported information about occupational and recreational UV exposure from questionnaires using validated questions [30].

We constructed a new variable, a UV exposure index, to account for cumulative UV exposure from multiple exposure sources according to the following factors: “occupational sun exposure (April–September)”, “recreational sun travel” and “sunbed use”. Respondents were considered to have high UV exposure if they provided ≥ 1 positive response in the high exposure category for any of these factors; medium UV exposure if they did not provide a positive response to any of the high categories and ≥ 1 response in a medium category; and low UV exposure if they provided positive response only in the lowest categories. For details on the construction of the UV exposure index, see Table S2.

We assumed that skin characteristics might affect an individual’s inclination to get a tattoo, and fair skin is an established risk factor for cSCC [31]. To investigate the association between tattoo exposure and cSCC among participants with such genetic risk factors for cSCC, we created a phenotypic risk index (high risk/medium risk/low risk) according to the Fitzpatrick scale [32], with skin pigmentation (Fitzpatrick skin types I-VI) and skin reaction after sun exposure (derived from Fitzpatrick skin types I-IV). Participants who reported “skin reaction to first sun exposure: burns (sometimes with blisters) and pain for at least 48 h” or “burns with pain that disappears within 24 h”, regardless of their skin tone, were classified as high risk. Participants who reported “skin reaction to first sun exposure: first burn, then tan”, regardless of skin tone, were classified as medium risk. Participants who reported a “very fair” or fair” skin tone and reported “skin reaction to first sun exposure: tan immediately” were classified as medium risk. Participants who reported “medium”, “olive”, “brown” or “dark brown” skin tone and reported “skin reaction to first sun exposure: tan immediately” were classified as low risk.

Although the direction of the association is unclear, SES seems to be related to cSCC [33–35] and is also associated with the prevalence of tattoos [36, 37]. We retrieved information about respondents’ educational attainment at the index date (categorized as primary and lower secondary/upper secondary/postsecondary education) and primary income source the year before the index date (categorized as [employed, student, parental leave, care of ill child or relative]/[unemployed, retired, sick leave, no income, financial aid]) from registers. We reasoned that the primary income source the year before diagnosis would better represent the SES because a cancer diagnosis may lead to sick leave and a lower income. Self-reported information about smoking (a proxy for SES) was obtained via the questionnaire; smoking was categorized as never smoker/ex-smoker/current smoker.

We hypothesized that lifelong diseases requiring immunosuppressive therapy, indicating a more severe stage of disease, may confound the association between tattoos and cSCC, particularly in the case of inflammatory skin diseases, such as psoriasis. With the background of the Koebner phenomenon, whereby insults to the skin such as tattoos can trigger, for example, psoriasis [38], we hypothesized that inflammatory skin disease may affect an individual’s likelihood of getting a tattoo. Patients on immunosuppressive medication, such as patients with psoriasis or patients with a transplanted organ, have a higher risk for cSCC [39, 40].

### Data sources

#### Registry data

Cases were identified in the National Cancer Register (National Board of Health and Welfare). Health care providers in Sweden are obliged to report all primary neoplasms using both clinical and morphological diagnostic codes, and the register coverage of skin cancers is 99% [41]. In addition, 99% of skin tumours are confirmed morphologically [42]. Statistics Sweden identified controls in the Total Population Register, and if patients were deceased, their next-of-kin was identified in the Total Population Register or the Multi-Generation Register. The National Patient Register (National Board of Health and Welfare) was used to exclude individuals at risk of psychological distress from participation and to retrieve data on conditions associated with immunosuppression. The National Prescribed Drug Register (National Board of Health and Welfare) was used to retrieve data on prescribed and filled immunosuppressive medication. Information about SES, including income source and education level, was retrieved from the Longitudinal Integrated Database for Health Insurance and Labor Market Studies (LISA, Statistics Sweden).

#### Questionnaire

A questionnaire designed to assess exposure to tattoos and other lifestyle factors was sent to the participants. The questionnaire has been described in detail elsewhere [7]. The next-of-kin of deceased individuals received a shorter questionnaire with less detailed questions. We piloted the questionnaire to assess the reliability of the exposure assessment by having 28 tattooed individuals assess their tattooed body surface on the same occasion as a trained research assistant. The weighted Cohen’s kappa for the ordinal variable was 0.79 [7]. The overall questionnaire was reviewed for content and clarity by (a) participants in the pilot study and (b) survey experts at Statistics Sweden.

### Study size

We used an a priori statistical power analysis to determine the sample size. We considered tattoo status as a dichotomous variable and assumed the tattoo prevalence to be 17% among the controls, as reported in the Swedish environmental health report in 2017 [6]. We wanted to be able to detect a risk ratio of 1.3 with 80% power. To achieve this, we would have to include 1300 cases with 2 controls per case. To account for the relatively low response rate of questionnaires, we aimed to include 3000 cases with 3 controls per case.

### Statistical methods

We used non-parametric statistics for descriptive analysis because our variables did not follow a normal distribution. We had few missing data and decided to perform complete case analyses.

We used logistic regression models to investigate the potential association between tattoos and cSCC. In a case-control study with incidence-density sampling, the odds ratio offers an unbiased estimate of the incidence rate ratio (IRR). To align with study protocol, we used conditional logistic regression in our primary analysis. To maximize the use of the available data and optimize precision, we also ran analyses using unconditional logistic regression, where we broke matching sets [43, 44]. Because we introduced selection bias by using balanced matching (case: control ratio 1:3) we made basic adjustments for age (5-year categories from < 40 years) with a term for residual age and sex to account for the original confounding and the introduced selection bias [45]. We also adjusted for time (index year in quartiles) in unconditional analysis to account for the bias introduced by breaking the matching [45]. The fully adjusted models were in addition to basic adjustments adjusted for education level, primary income source, smoking, phenotypic risk index and UV exposure index. We investigated potential effect modification by UV exposure by including an interaction term (tattoo × UV exposure index) in the model. For the interaction analysis we merged the UV-index categories “low” and “medium” for sufficient statistical power. A potential crude dose-response relationship between the area of tattooed body surface and cSCC was investigated. We categorized the tattooed body surface as < 1 palm;>1 palm; no tattoo, and number of sessions as 1 session;2–3 sessions;>4 sessions.

### Sensitivity analysis

We limited the cases to individuals receiving their first ever cSCC diagnosis in a sensitivity analysis. Secondly, we explored the potential modifying role of immunosuppression on the association between tattoo exposure and cSCC in the fully adjusted model by considering the use of immunosuppressive drugs (a) an effect modifier by inclusion of an interaction term, and (b) a confounder by inclusion of a fixed effect. We used registry data on ≥ 1 filled prescription of immunosuppressive drugs before the index date (i.e. ATC code L04). Finally, the next-of-kin of deceased individuals (*n* = 19) and their controls (*n* = 206) were included in a sensitivity analysis, where UV exposure index was based only on questions included in the questionnaire sent to relatives: “sunbed use” and “occupational sun exposure (April–September)”, and phenotypic risk index were not included.

## Results

### Study population

**Fig. 1:**
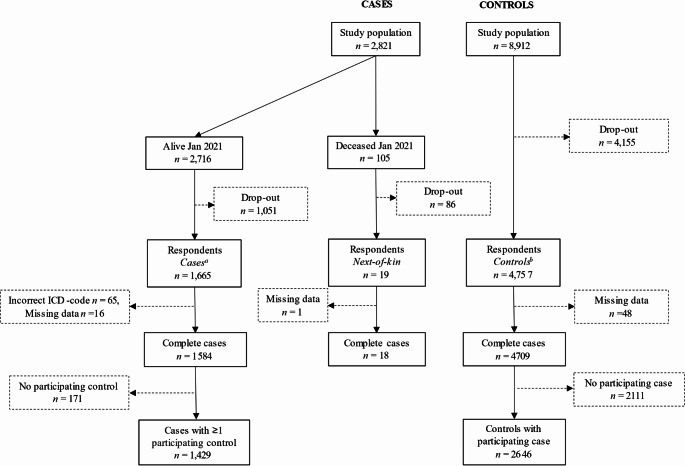
Flowchart of cases, controls and next-of-kin from the sampling frame to the statistical analysis. All available cases aged 20–60 years during 2014–2017 were included in the sampling frame. ^a^Number of respondents included in crude unmatched analysis were *n* = 1600, due to the 65 incorrect ICD-codes. ^b^Number of respondents included in crude unmatched analysis were *n* = 4551. The remaining *n* = 206 controls were from risk sets with deceased cases and therefore only included in the next-of-kin sensitivity analysis

In January 2021, 95% (*n* = 2716) of the identified cases were alive (Fig. 1). A total of 1665 cases, 4757 controls, and 19 next-of-kin responded to the questionnaire, giving response rates of 61%, 53% and 16%, respectively.

### Dropout analysis

The descriptive dropout analysis (Table S3) showed that respondents were more often women, older, married, of higher SES, with a higher level of education, and born in Sweden. We also found that responders among the cases had slightly higher income and education levels and were born in Sweden to a greater extent than the responders among the controls.

### Descriptive characteristics

The median age was 56 years (interquartile range, 9 years) for the cases and 56 years (interquartile range, 10 years) for the controls. Participating cases and controls were fairly similarly distributed with regard to sex, educational attainment and marital status. Cancer cases had a higher household disposable income than controls. Cancer cases had a lower frequency of current smokers than the control group, but a higher proportion of previous smokers (Table 1).

**Table 1:** Characteristics of the participating cases and controls

	Cases, *n* (%)	Controls, *n* (%)
Sex		
Male	653 (41)	1756 (39)
Female	947 (59)	2795 (61)
Educational attainment, index date		
Primary and lower secondary	111 (7)	407 (9)
Upper secondary	683 (43)	2073 (46)
Postsecondary	804 (50)	2060 (45)
Missing	2	11
Disposable income, household (SEK)		
< 340,800	353 (22)	1135 (25)
340,800–538,200	364 (23)	1136 (25)
538,300–737,500	392 (25)	1137 (25)
≥ 737,600	488 (31)	1137 (25)
Missing	3	6
Marital status		
Married/registered partnership	925 (58)	2665 (59)
Divorced/widowed	305 (19)	780 (17)
Unmarried	369 (23)	1105 (24)
Missing	1	1
Smoking		
Yes, current	120 (8)	516 (11)
Yes, previously	621 (39)	1583 (35)
No, never	854 (54)	2447 (54)
Missing	5	5

Cases were more exposed to UV radiation from recreational exposure such as holidays in sunny destinations and sunbeds, and thus had a higher frequency in the highly exposed category of the UV exposure index (Table 2). Unexpectedly, cases were working outside to a lesser extent than controls, but the proportion of time outside for those who worked outside was similar between the groups.

**Table 2:** Self-reported UV exposure at the time of the survey

	Cases, *n* (%)	Controls, *n* (%)
Occupational sun exposure (April–September)		
Yes	255 (16)	945 (21)
No	1078 (68)	2860 (63)
I don’t work	261 (16)	724 (16)
Missing	6	22
Occupational sun exposure (April–September), time outside		
>20 h/week	67 (25)	292 (29)
6–20 h/week	125 (47)	450 (45)
≤5 h/week	74 (28)	253 (25)
Recreational sun travel < 13 years of age		
Yes, every year	397 (25)	851 (19)
Yes, occasionally	1005 (63)	2882 (64)
No, never	183 (12)	786 (17)
Missing	15	32
Sunbed use > 13 years of age		
Several times per year	355 (22)	587 (13)
Occasionally	888 (56)	2462 (54)
Never	349 (22)	1476 (33)
Missing	8	26
UV exposure index		
Ever high exposure	656 (41)	1486 (32)
Never high exposure, ever medium exposure	871 (55)	2697 (59)
Never high exposure, never medium exposure	67 (4)	362 (8)
Missing	6	6

**Table 3:** Phenotypic characteristics of the cases and controls

	Cases, *n* (%)	Controls, *n* (%)
Natural eye colour		
Blue	883 (56)	2143 (47)
Grey	32 (2)	138 (3)
Green	138 (9)	485 (11)
Yellow	0 (0)	5 (0.1)
Light brown	73 (5)	283 (6)
Dark brown	88 (6)	439 (10)
Mixed (green, blue, grey)	369 (23)	1000 (22)
Different coloured eyes	6 (0.4)	32 (0.7)
Missing	11	26
Natural skin tone		
Very light	192 (12)	196 (4)
Light	911 (58)	2287 (51)
Medium	461 (29)	1890 (42)
Olive/light brown	16 (1)	100 (2)
Brown	2 (0.1)	35 (0.8)
Dark brown	1 (0.1)	5 (0.1)
Missing	17	38
Freckles after sun exposure		
Yes, many	274 (17)	357 (8)
Yes, a few	570 (36)	1358 (30)
No, I don’t get freckles	745 (47)	2818 (62)
Missing	11	18
Skin reaction to first sun exposure		
Burns (sometimes with blisters) and pain/sting for at least 48 h	45 (3)	79 (2)
Burns with pain/sting that disappears within 24 h	596 (37)	1163 (26)
Initially burns, then tans	855 (54)	2783 (62)
Tan immediately	95 (6)	494 (11)
Missing	9	32
Childhood eczema		
Yes	310 (20)	754 (17)
No	1108 (70)	3241 (72)
I don’t know	170 (11)	527 (12)
Missing	12	29
Phenotypic risk index		
High risk	644 (40)	1258 (28)
Medium risk	885 (55)	2890 (64)
Low risk	69 (4)	401 (9)
Missing	2	2
Immunosuppressive therapy		
Yes	235 (15)	126 (3)
No	1375 (85)	4425 (97)

Cases were to a higher extent blue-eyed with a very fair skin type, as is to be expected for cSCC as fair skin is a risk factor, and many more reported freckles after sun exposure (Table 3). More cases than controls stated that they had eczema during childhood. Cases were less prone to tan immediately after sun exposure; rather their skin burned to a higher extent, reflecting the higher proportion of participants with fair skin among cases. Cases were overrepresented in the high-risk category of the phenotypic risk index. 15% (*n* = 235) of the cases and 3% (*n* = 126) of the controls received immunosuppressive therapy before the index date.

The cases were slightly less tattooed than the controls (15% vs. 18%; Table 4). The median age at first tattoo was 30 years for both cases and controls, and the area of tattooed body surface and the choice of colours were similar. Cases stated that they sunbathed less after tattooing or covered their tattoos while sunbathing, to a higher extent than controls. The majority of cases (76%) and controls (84%) had not changed their tanning habits after getting tattooed.

**Table 4:** Tattoo characteristics among tattooed cases and controls

Tattoo characteristics	Cases, *n* (%)	Controls, *n* (%)
Tattoo		
Yes	236 (15)	802 (18)
No	1364 (85)	3749 (82)
Missing	0	0
Tattoo type		
Decorative	220 (93)	753 (94)
Cosmetic	22 (9)	53 (7)
Medical	11 (5)	30 (4)
Missing	14	31
Size of tattoo		
>5 palm	14 (6)	52 (7)
1—5 palms	76 (32)	262 (33)
< 1 palm	146 (62)	481 (60)
Missing	0	7
Colour of tattoo		
Only black/grey	81 (34)	271 (34)
Both black/grey and colour	107 (45)	393 (49)
Only colour	48 (20)	135 (17)
Missing	0	3
Number of sessions		
1	100 (42)	384 (48)
2–3	88 (37)	261 (33)
4–5	28 (12)	78 (10)
6–9	9 (4)	44 (6)
>=10	11 (5)	29 (4)
Missing	0	6
Tattooist		
Professional, studio	192 (81)	662 (83)
Professional, other facility	31 (13)	96 (12)
Cosmetic tattooist, in studio or clinic	20 (8)	38 (5)
Healthcare professional, in clinic	12 (5)	29 (4)
Nonprofessional, irrespective of location	18 (8)	72 (9)
Missing	0	0
Geographical region^b^		
Sweden	203 (86)	687 (86)
Nordics, except Sweden	25 (11)	91 (11)
Rest of Europe	15 (6)	48 (6)
Asia	14 (6)	39 (5)
Oceania	2 (1)	1 (0.001)
USA	7 (3)	17 (2)
Other	3 (1)	18 (2)
Missing	0	0
Changed tanning habits since first tattoo		
Yes, I sunbathe more	0 (0)	5 (0.6)
Yes, I sunbathe less	30 (13)	51 (6)
Yes, I sunbathe as before, but cover tattooed skin or protect it with SPF*	25 (11)	75 (9)
No	179 (76)	669 (84)
Missing	2	2

*Sun protection factor

### Anatomical location of tattoos and cutaneous squamous cell carcinoma

Among 78% (*n* = 194) of the tattooed cases, we had information about the side and anatomical location of the malignancy. Among 23% of those cases, the tattoo was placed on the same anatomical location as the cancerous lesion, although not statistically correlated (Spearman’s rho = 0.04, p-value = 0.58).

### Tattoos and cutaneous squamous cell carcinoma

We did not find an increased risk for cSCC among tattooed individuals. The point estimates did not indicate an increased risk (IRR = 0.95; 95% CI, 0.78–1.15; Table 5), but in unmatched analysis, they were suggestive of lower risk (IRR = 0.88; 95% CI, 0.74–1.04) although the CI included 1. We did not find evidence of effect modification by UV exposure (overall P[interaction] = 0.65; Table S4). Neither did we find any evidence for effect modification by immunosuppressive drugs (overall P[interaction] = 0.47; Table S5), and we did not find any risk increase when adjusting for immunosuppressive drugs or for different exposure durations, or when including *next-of-kin* of deceased cases (Table 5 and Table S6).

**Table 5:** Basic and fully adjusted incidence rate ratios (IRRs) of cutaneous squamous cell carcinoma in tattooed individuals relative to non-tattooed individuals

	Matched						Unmatched					
	Basic adjustment^a^			Fully adjusted^b^			Basic adjustment^c^			Fully adjusted^d^		
	Cases, *n*	Controls, *n*	IRR (95% CI)	Cases, *n*	Controls, *n*	IRR (95% CI)	Cases, *n*	Controls, *n*	IRR (95% CI)	Cases, *n*	Controls, *n*	IRR (95% CI)
**Main analyses**												
*Tattoo status*	1429	2609		1415	2595		1600	4551		1584	4526	
Tattooed	213	418	0.88 (0.74–1.06)	211	414	0.95 (0.78–1.15)	236	802	0.83 (0.71–0.97)	234	798	0.88 (0.74–1.04)
Non-tattooed	1216	2191	1.00	1204	2181	1.00	1364	3749	1.00	1350	3728	1.00
*Tattooed body surface*	1429	2607		1415	2593		1600	4544		1544	4519	
Tattooed body surface > 1 palm	79	145	0.92 (0.69–1.23)	78	145	0.98 (0.72–1.34)	90	314	0.82 (0.64–1.05)	89	314	0.85 (0.66–1.10)
Tattooed body surface < 1 palm	134	271	0.87 (0.70–1.09)	133	267	0.93 (0.74–1.17)	146	481	0.85 (0.70–1.03)	145	477	0.88 (0.72–1.08)
Non-tattooed	1216	2191	1.00	1204	218	1.00	1364	3749	1.00	1350	3728	1.00
*Ordinal exposure variable*	1429	2607		1415	2593		1600	4545		1584	4520	
1 tattoo session	92	215	0.76 (0.59–0.98)	92	211	0.83 (0.63–1.08)	100	384	0.73 (0.58–0.92)	100	380	0.77 (0.61–0.98)
2–3 tattoo sessions	80	133	1.06 (0.78–1.43)	79	133	1.07 (0.78–1.47)	88	261	0.95 (0.74–1.22)	87	261	0.98 (0.76–1.27)
>4 tattoo sessions	41	68	1.00 (0.67–1.49)	40	68	1.12 (0.73–1.73)	48	151	0.93 (0.66–1.29)	47	151	1.02 (0.72–1.44)
Non-tattooed	1216	2191	1.00	1204	2181	1.00	1364	3749	1.00	1350	3728	1.00
*Colour*	1429	2608		1415	2594		1600	4548		1584	4523	
Black/grey	71	138	0.90 (0.67–1.22)	70	136	0.97 (0.71–1.33)	81	271	0.86 (0.68–1.12)	80	269	0.92 (0.70–1.20)
Black/grey + colour	98	201	0.85 (0.66–1.10)	97	199	0.91 (0.69–1.20)	107	393	0.77 (0.61–0.96)	106	391	0.80 (0.63-1.00)
Colour only	44	78	0.96 (0.66–1.41)	44	78	1.01 (0.67–1.51)	48	135	0.96 (0.69–1.35)	48	135	0.98 (0.69–1.38)
No tattoo	1216	2191	1.00	1204	2181	1.00	1364	3749	1.00	1350	3728	1.00
*Exposure duration*	1424	2595		1410	2581		1594	4530		1578	4505	
0–5 years	22	46	0.91 (0.54–1.53)	22	46	0.90 (0.52–1.54)	22	84	0.75 (0.47–1.21)	22	84	0.78 (0.48–1.26)
6–10 years	21	40	0.94 (0.55–1.63)	21	38	1.18 (0.67–2.10)	22	92	0.70 (0.43–1.12)	22	90	0.81 (0.50–1.31)
11–15 years	25	56	0.79 (0.48–1.29)	25	56	0.85 (0.52–1.41)	30	106	0.83 (0.55–1.25)	30	106	0.84 (0.55–1.27)
> 15 years	140	262	0.90 (0.72–1.12)	138	260	0.96 (0.75–1.22)	156	499	0.87 (0.72–1.06)	154	497	0.92 (0.75–1.13)
Non-tattooed	1216	2191	1.00	1204	2181	1.00	1364	3749	1.00	1350	3728	1.00
*Restriction: first ever diagnosis only*	1204	2609		1192	2595		1357	4551		1343	4526	
Tattooed	187	418	0.94 (0.77–1.15)	185	414	1.04 (0.84–1.29)	208	802	0.86 (0.73–1.02)	206	798	0.92 (0.77–1.10)
Non-tattooed	1017	2191	1.00	1007	2181		1149	3749	1.00	1137	3728	1.00
*Sensitivity analysis: immunosuppressive drugs*	1429	2609		1415	2595		1600	4551		1584	4526	
Tattooed	213	418	0.88 (0.74–1.06)	211	414	0.97 (0.79–1.20)	236	802	0.83 (0.71–0.97)	234	798	0.88 (0.74–1.04)
Non-tattooed	1216	2191	1.00	1204	2181	1.00	1364	3749	1.00	1350	3728	1.00

^a^Estimates obtained from conditional regression models adjusted for sex and age.

^b^Estimates obtained from conditional regression models adjusted for age, sex, educational attainment, household disposable income, marital status, phenotypic risk index, UV-exposure index, and smoking.

^c^Estimates obtained from unconditional regression models adjusted for sex, age, and index year.

^d^Estimates obtained from unconditional regression models adjusted for age, sex, index year, educational attainment, household disposable income, marital status, phenotypic risk index, UV-exposure index, and smoking.

## Discussion

In this study of tattoos as a possible risk factor for squamous cell carcinoma, we found no evidence of an association. In the unmatched analysis, we found that tattooed individuals had a slightly lower risk than non-tattooed individuals in contrast to our hypothesis that a tattoo could be a risk factor for cSCC. However, confidence intervals (CIs) were wide and included 1, and in combination with the matched results we interpret this as a null result.

We assumed that UV exposure was a confounder for the association between tattoo exposure and cSCC through an unmeasured common cause reflecting a general interest in one’s appearance. Moreover, we expected UV exposure to modify the effect of the association between tattoos and cSCC, because of the possible release of potentially carcinogenic aromatic amines from azo pigments in the tattoo when exposed to UV radiation [15, 16]. However, we found no evidence of UV exposure as an effect modifier of the association between tattoos and cSCC, but further investigation is warranted for this and other skin malignancies in settings with higher UV exposure, because of the relatively low level of UV exposure in our setting. The analysis using exposure duration as exposure confirmed that too short latency periods did not mask an association with cSCC. Excluding individuals with immunosuppressive therapy did not affect the results. 15% of cases versus 3% of controls were on immunosuppressive therapy, indicating that the case group may have worse overall health than controls; this perhaps explains why cases were tattooed to a lesser extent than controls. We did not find that the results changed when including next-of-kin, although they were very few. We can therefore not draw any conclusions on survivorship bias in this study. The robust effect estimate suggests that the results were not sensitive to analytical decisions.

### Strengths and limitations

This is the first study to address tattoos as a risk factor for cSCC and it includes a large population-based sample with sufficient statistical power to detect associations; 51% of invited participants replied to the questionnaire. We have access to National Administrative Registers with full population coverage and valid data on both the outcome and sociodemographic confounders [46, 47]. Thus, we have a low risk of loss-to-follow up, and with that a low risk for information bias and a low risk for selection bias with respect to register data [48]. The access to registers also facilitates the study of rare outcomes, which would be more difficult in longitudinal cohort studies where the inclusion of enough cases is a limitation.

We found higher frequencies of conventional risk factors for cSCC among cases, such as high UV exposure and fair skin and we did not find this same pattern for tattoos.

To reduce the risk of selection bias, the study population was informed that the purpose of the study was to investigate associations between several lifestyle factors on multiple outcomes to avoid conditioning participation based on both exposure and outcome status. We consider the risk of recall bias regarding the exposure variable to be low. According to the dropout analysis, respondents were of higher SES and more often born in Sweden. Additionally, response rates were higher among cases than controls. As both lower SES and occupational UV exposure from bluecollar work likely are associated with a higher risk for cSCC as well as with a higher tattoo prevalence, our results may be an underestimation of the true effect. Making causal assumptions based on the results of one single study is premature and it remains to be seen if our results can be replicated by further research.

One limitation is that some of our data, including information about one confounding factor (UV exposure) are based on self-reported information. There is always a risk for recall bias, and in this study, there was a risk that cases over-reported their sun exposure because it is a well-known risk factor for cSCC. However, recall bias only seemed to have a minor effect on risk estimates in a large nested case-control study of skin cancer [49, 50]. The authors did find minor indications of recall bias but patterns were inconsistent, and they did not observe recall bias for sun bed use, and other UV radiation related exposures, the variables most relevant for the UV exposure index in the present study.

The proportion of deceased cases was low, and we therefore consider the risk of survivorship bias to be low. Nonetheless, we tried to avoid survivorship bias by including answers from relatives of deceased individuals; however, this analysis was of limited value due to a low participation rate among next-of-kin.

Our study focused on cSCC developing in mid-life. Thus, the results may not be generalizable to older populations because the risk factors may be different. In addition, we cannot generalize our results to more UV-intense regions of the world. Also, our cases and controls came from a Nordic country with limited variation in skin pigmentation. Populations with melanin-rich skin are not at high risk for developing UV-related cSCC; human papillomavirus is a bigger risk factor [51]. Hence, the association between tattoos and cSCC may be different in other populations with melanin-rich skin.

## Conclusion

This study of tattoos as a risk factor for cSCC found no evidence of an association. However, more epidemiologic studies are needed before consensus regarding a lack of association can be reached. In addition, the results cannot be extrapolated to other malignancies and further research is warranted to clarify the role of tattoos as a risk factor for other cancer types.

## Electronic supplementary material

Below is the link to the electronic supplementary material.

Supplementary Material 1

## Data Availability

The data presented in this study are not publicly available due to privacy and legal restrictions (GDPR). Data sharing is not possible.
