# Supplementary material for: Tattoos and cutaneous squamous cell carcinoma: a population-based case-control study
Source: Eur J Epidemiol. 2025 Apr 25;40(4):451–61. doi: 10.1007/s10654-025-01230-z (PMC12145309; doi:10.1007/s10654-025-01230-z)
Supplement: Supplementary file 1 — Supplementary Material 1 [file 10654_2025_1230_MOESM1_ESM.docx]

Supplementary data

Is tattoos a risk factor for cutaneous squamous cell carcinoma? A population-based case-control study

Emelie Rietz Liljedahl^1^, Malin Engfeldt^1,2^, Kari Nielsen^3,4^, Anna Jöud^1,5^, and Christel Nielsen^1,6^

^1^Division of Occupational and Environmental Medicine, Department of Laboratory Medicine, Lund University, Lund, Sweden

^2^Department of Occupational and Environmental Medicine, Region Skåne, Lund, Sweden

^3^ Department of Dermatology, Skåne University Hospital, Lund, Sweden

^4^Dermatology, Department of Clinical Sciences, Lund University, Lund, Sweden

^5^Department of Clinical Sciences, Lund University, Lund, Sweden

^6^Clinical Pharmacology, Pharmacy and Environmental Medicine, Institute of Public Health, University of Southern Denmark, Odense, Denmark

Table S1. Morphology types and corresponding morphology codes (SNOMED; Systematized Nomenclature of Medicine) according to the International Classification of Diseases for Oncology, 3rd Edition

| Morphology type | SNOMED3 | Number |
| --- | --- | --- |
| Squamous cell carcinoma with osteoclast-like giant cells | 80353 |  |
| Squamous cell carcinoma, verrucous type | 80513 | 2 |
| Squamous cell carcinoma in situ | 80702/b | 679 |
| Squamous cell carcinoma | 80703 | 526 |
| Squamous cell carcinoma, keratoacanthoma type | 80713 | 1 |
| Squamous cell carcinoma, pseudovascular/sarcomatoid type/spindle cell type | 80743 |  |
| Squamous cell carcinoma, acantholytic type | 80753 |  |
| Squamous cell carcinoma in situ, Morbus Bowen | 80812 | 392 |
| Lymphoepithelioma/lymphoepithelioma like carcinoma/lymphoepithelial carcinoma | 80823 |  |
| Squamous cell carcinoma, clear cell type | 80843 |  |
| Adenosquamous carcinoma | 85603 |  |

Table S2. Items (columns) and categories (rows) used for the construction of the UV exposure index.

| UV exposure index | Occupational sun exposure | Recreational sun travel | Sunbed use |
| --- | --- | --- | --- |
| High | More than 20 hours per week | Every year after 13 years of age | Several times per year |
| Medium | 6-20 hours per week | Occasionally | Occasionally |
| Low | No, or max 5 hours per week | Never | Never |

Categories under each factor represent questionnaire alternatives. Respondents were considered to have high UV exposure if they provided ≥1 positive response in the high exposure category for any of these factors; medium UV exposure if they did not provide a positive response to any of the high categories and ≥1 response in a medium category; and low UV exposure if they provided positive response only in the lowest categories.

Table S3. Demographics of respondents and dropouts by outcome status

|  | Cases | | | | Controls | | | |
| --- | --- | --- | --- | --- | --- | --- | --- | --- |
|  | Respondents | | Dropouts | | Respondents | | Dropouts | |
|  | No. | % | No. | % | No. | % | No. | % |
| Gender |  |  |  |  |  |  |  |  |
| Men | 684 | 41 | 514 | 44 | 1853 | 39 | 1962 | 47 |
| Women | 981 | 59 | 659 | 56 | 2905 | 61 | 2192 | 53 |
| Age, 10-year intervals |  | |  | |  | |  | |
| 20–29 years | 3 | 0.2 | 5 | 0.4 | 8 | 0.2 | 22 | 0.5 |
| 30–39 years | 19 | 1.1 | 31 | 2.6 | 55 | 1.2 | 96 | 2.3 |
| 40–49 years | 92 | 5.5 | 137 | 12 | 318 | 6.7 | 390 | 9.4 |
| 50–59 years | 586 | 35 | 486 | 41 | 1656 | 35 | 1668 | 40 |
| 60–69 years | 965 | 58 | 514 | 44 | 2721 | 57 | 1978 | 48 |
| Country of birth |  | |  | |  | |  | |
| Sweden | 1537 | 92 | 1018 | 87 | 4168 | 88 | 3061 | 74 |
| Outside Sweden | 128 | 7.7 | 155 | 13 | 590 | 12 | 1093 | 26 |
| Citizenship |  | |  | |  | |  | |
| Swedish | 1628 | 98 | 1146 | 98 | 4643 | 98 | 3910 | 94 |
| Foreign | 37 | 2.2 | 27 | 2.3 | 115 | 2.4 | 244 | 5.9 |
| Civil status |  | |  | |  | |  | |
| Married | 978 | 59 | 578 | 49 | 2747 | 58 | 1936 | 47 |
| Not married | 355 | 21 | 334 | 29 | 1108 | 23 | 1187 | 29 |
| Divorced | 296 | 18 | 244 | 21 | 796 | 17 | 929 | 22 |
| Widowed | 36 | 2.2 | 17 | 1.4 | 107 | 2.2 | 102 | 2.5 |
| Income |  | |  | |  | |  | |
| 0 SEK | 31 | 1.9 | 33 | 2.8 | 97 | 2 | 209 | 5 |
| 1–124 999 SEK | 63 | 3.8 | 76 | 6.5 | 175 | 3.7 | 394 | 9.5 |
| 125 000–199 999 SEK | 81 | 4.9 | 123 | 11 | 290 | 6.1 | 390 | 9.4 |
| 200 000–279 999 SEK | 159 | 9.5 | 115 | 9.8 | 500 | 11 | 571 | 14 |
| 280 000–369 999 SEK | 323 | 19 | 259 | 22 | 1112 | 23 | 1005 | 24 |
| ≥370 000 SEK | 1008 | 61 | 567 | 48 | 2584 | 54 | 1585 | 38 |
| Education |  | |  | |  | |  | |
| Primary- and lower secondary school | 114 | 6.8 | 137 | 12 | 422 | 8.9 | 770 | 19 |
| Upper-secondary school | 711 | 43 | 566 | 48 | 2160 | 45 | 2122 | 51 |
| Post-secondary education, < 3 years | 317 | 19 | 204 | 17 | 855 | 18 | 518 | 13 |
| Post-secondary education, 3 years - | 521 | 31 | 263 | 22 | 1311 | 28 | 687 | 17 |
| Unknown | 2 | 0.1 | 3 | 0.3 | 10 | 0.2 | 57 | 1.3 |

Table S4. Adjusted incidence rate ratios of cutaneous squamous cell carcinoma in tattooed and non-tattooed individuals with different extents of sun exposure, expressed in relation to non-tattooed individuals with low UV exposure from interaction analysis.

|  | Matched^a^ | | | Unmatched^b^ | | |
| --- | --- | --- | --- | --- | --- | --- |
| Interaction | Cases  (*n*) | Controls  (*n*) | IRR (95% CI) | Cases  (*n*) | Controls  (*n*) | IRR (95% CI) |
| High UV, tattooed | 109 | 161 | 1.54 (1.17-2.04) | 120 | 332 | 1.33 (1.05-1.69) |
| High UV, non-tattooed | 481 | 652 | 1.54 (1.31-1.80) | 531 | 1149 | 1.50 (1.31-1.72) |
| Low-medium UV, tattooed | 102 | 253 | 0.91 (0.71-1.19) | 114 | 466 | 0.89 (0.71-1.12) |
| Low-medium UV, non-tattooed | 723 | 1529 | 1.00 | 819 | 2579 | 1.00 |

^a^Estimates obtained from conditional regression models adjusted for sex, age, educational attainment, household disposable income, marital status, UV exposure and smoking, with an interaction term for tattoos and UV exposure.

^b^Estimates obtained from unconditional regression models adjusted for sex, age, index year, educational attainment, household disposable income, marital status, UV exposure and smoking, with an interaction term for tattoos and UV exposure.

Table S5. Adjusted incidence rate ratios of cutaneous squamous cell carcinoma in tattooed and non-tattooed individuals with different status for immunosuppressive drugs, expressed in relation to non-tattooed individuals with no immunosuppressive drugs from interaction analysis.

|  | Matched^a^ | | | Unmatched^b^ | | |
| --- | --- | --- | --- | --- | --- | --- |
| Interaction | Cases  (*n*) | Controls  (*n*) | IRR (95% CI) | Cases  (*n*) | Controls  (*n*) | IRR (95% CI) |
| Immunosuppressive meds, tattooed | 30 | 15 | 4.93 (2.52-9.63) | 36 | 27 | 5.16 (3.07-8.67) |
| Immunosuppressive meds, non-tattooed | 178 | 68 | 6.57 (4.78-9.03) | 197 | 96 | 7.30 (5.62-9.48) |
| No immunosuppressive meds, tattooed | 181 | 399 | 0.99 (0.80-1.23) | 198 | 771 | 0.89 (0.75-1.07) |
| No immunosuppressive med, non-tattooed | 1026 | 2113 | 1.00 | 1153 | 3632 | 1.00 |

^a^Estimates obtained from conditional regression models adjusted for sex, age, educational attainment, household disposable income, marital status, UV exposure, immunosuppressive drug status and smoking, with an interaction term for tattoos and immunosuppressive drugs.

^b^Estimates obtained from unconditional regression models adjusted for sex, age, index year, educational attainment, household disposable income, marital status, UV exposure, immunosuppressive drug status and smoking, with an interaction term for tattoos and immunosuppressive drugs.

Table S6. Crude and adjusted incidence rate ratios (IRRs) of cutaneous squamous cell carcinoma in tattooed individuals including next-of-kin relative to non-tattooed individuals

| Sensitivity analysis | Matched | | | Unmatched | | |
| --- | --- | --- | --- | --- | --- | --- |
|  | Fully adjusted^a^ | | | Fully adjusted^b^ | | |
|  | Cases (*n*) | Controls (*n*) | IRR (95% CI) | Cases (*n*) | Controls (*n*) | IRR (95% CI) |
| Including next-of-kin | 1433 | 2633 |  | 1603 | 4705 |  |
| Tattooed | 211 | 420 | 0.91 (0.75-1.10) | 235 | 829 | 0.85 (0.72–1.00) |
| Non-tattooed | 1222 | 2213 | 1.00 | 1368 | 3876 | 1.00 |

CI, confidence interval.

^a^Estimates obtained from conditional regression models adjusted for sex, age, educational attainment, household disposable income, marital status, UV exposure and smoking.

^b^Estimates obtained from unconditional regression models adjusted for sex, age, index year, educational attainment, household disposable income, marital status, UV exposure and smoking*^.^*


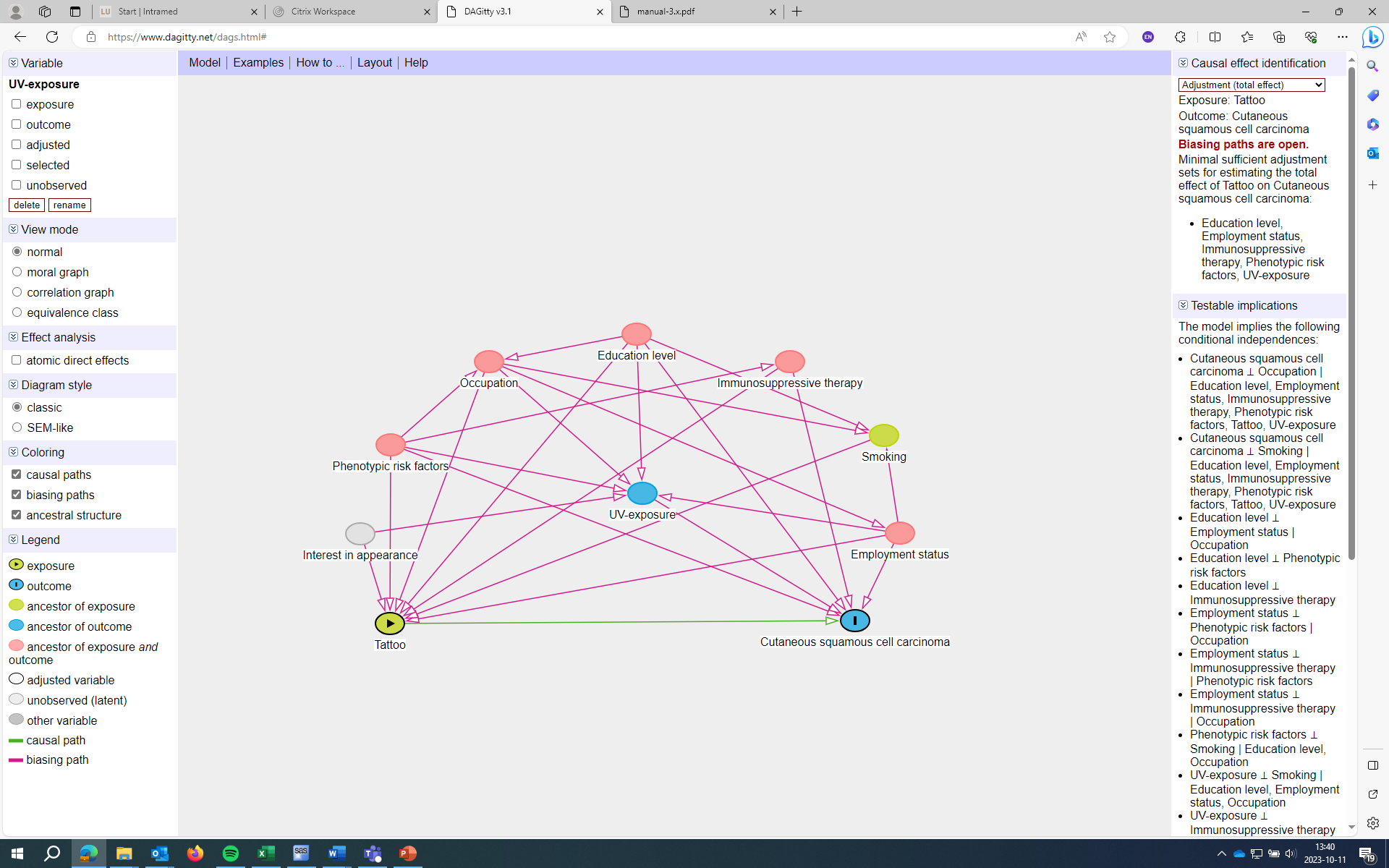


Figure S1. Directed acyclic graph of the association between tattoos and cutaneous squamous cell carcinoma, including possible measurable and unmeasurable confounders.
